# Contaminant emissions as indicators of chemical elements in the snow along a latitudinal gradient in southern Andes

**DOI:** 10.1038/s41598-021-93895-1

**Published:** 2021-07-15

**Authors:** Jaime Pizarro, Pablo M. Vergara, Sergio Cerda, Raúl R. Cordero, Ximena Castillo, Penny M. Rowe, Gino Casassa, Jorge Carrasco, Alessandro Damiani, Pedro J. Llanillo, Fabrice Lambert, Roberto Rondanelli, Nicolas Huneeus, Francisco Fernandoy, Juan Alfonso, Steven Neshyba

**Affiliations:** 1 Universidad de Santiago de Chile (USACH), Santiago, Chile; 2 NorthWest Research Associates, Redmond, WA USA; 3 Universidad de Magallanes, Punta Arenas, Chile; 4 Center for Environmental Remote Sensing, Chiba University, Chiba, Japan; 5 Department of Physical Geography, Pontificia Universidad Católica de Chile, Santiago, Chile; 6 Universidad de Chile, Blanco Encalada 2002, Santiago, Chile; 7 Center for Climate and Resilience Research CR2, Blanco Encalada 2002, Santiago, Chile; 8 Universidad Nacional Andrés Bello, Viña del Mar 2531015, Valparaíso, Chile; 9 Instituto Venezolano de Investigaciones Científicas (IVIC), Carretera Panamericana, Km 11, Altos de Pipe, Venezuela; 10 Department of Chemistry, University of Puget Sound, Tacoma, WA USA

**Keywords:** Climate sciences, Environmental sciences

## Abstract

The chemical composition of snow provides insights on atmospheric transport of anthropogenic contaminants at different spatial scales. In this study, we assess how human activities influence the concentration of elements in the Andean mountain snow along a latitudinal transect throughout Chile. The concentration of seven elements (Al, Cu, Fe, Li, Mg, Mn and Zn) was associated to gaseous and particulate contaminants emitted at different spatial scales. Our results indicate carbon monoxide (CO) averaged at 20 km and nitrogen oxide (NOx) at 40 km as the main indicators of the chemical elements analyzed. CO was found to be a significant predictor of most element concentrations while concentrations of Cu, Mn, Mg and Zn were positively associated to emissions of NOx. Emission of 2.5 μm and 10 μm particulate matter averaged at different spatial scales was positively associated to concentration of Li. Finally, the concentration of Zn was positively associated to volatile organic compounds (VOC) averaged at 40 km around sampling sites. The association between air contaminants and chemical composition of snow suggests that regions with intensive anthropogenic pollution face reduced quality of freshwater originated from glacier and snow melting.

## Introduction

Gaseous and particulate matter contaminants emitted from industrialized areas are easily dispersed in the atmosphere, reaching remote regions worldwide such as the poles and high-altitude mountains^1–3^. These atmospheric particles act as dispersal vectors for elements, many of them potentially toxic to plants and animals, with a fraction of them being eventually deposited upon snow^4^. Once the polluted snow melts, depending on their characteristics (e.g., organic or inorganic), these particles can be transported downstream, thus contaminating water used for human consumption or farming^5,6^. Elements accumulated in the snow may eventually reach the sea, thus affecting ocean–atmosphere interaction in the global biogeochemical cycle^7,8^.

The chemical composition of snow provides insights on the air contaminants emitted in a geographic region^2^. However, the presence of potentially toxic elements in the snow can be originated from natural and anthropogenic sources^9^. The deposition rates and enrichment of potentially toxic elements in the snow can be addressed through different analyses, such as isotopic studies or crustal enrichment factors^9,10^. Elements present in snow may originate from different anthropogenic sources, including industrial pollution, urban emissions and dust carried by the wind^11^. Long-distance dispersal of elements derived from air pollution has been inferred from ice and snow samples collected from high-altitude mountains like the Andes and Himalayas^12^, as well as from remote zones of the Polar Regions^13^. Snowflakes intercept and capture atmospheric contaminants, which are temporally accumulated into the snow and subsequently transported downstream^13^. Indeed, a high load of chemical species are usually found on mountains surrounded by industrialized and urbanized areas, such as Andean mountains in central Chile^14,15^, the Eastern European Alps^16^, the Sichuan Basin in southwest China^17,18^, in Northwest China^12^, and in the Himalayan range^19^. The amount of contaminant emissions from industrial and urban lands depends on socio-economic factors characterizing each region^20^. The emission of pollutants not only impacts human health and ecosystems, but also contribute to the observed retreat of the Andean cryosphere^21^. Consequently, the load of chemical species in high-mountain snow may exhibit a certain degree of geographic variability, which could provide the basis for managing the provision of clean water for the surrounding populations.

In Chile, emission of particulate matter varies latitudinally in terms of the type and concentration of contaminants emitted^22^ and its dispersion depends on the regional and local meteorology. The prevailing circulation affecting the Andes Mountains is westerly airflow, although subsidence inversion and topography in northern and central Chile can originate local circulations within the near-surface atmospheric boundary^23^. In the northernmost regions (approx. 20°S–30°S), power plants, mining and the presence of arid soils are important factors contributing to the composition and abundance of elements^24,25^. Pollution sources are different in Central and Southern Chile. Most of the urban lands are concentrated in Central Chile^26^, while the intensive use of coal and biomass fuels in the southern regions is responsible for the high levels of atmospheric contamination found in these regions^27,28^. Snow in Central Chile is vulnerable to a combination of urban and mining pollution sources, as recently found for black carbon in the Andes Mountains near Santiago^29^. The near-surface westerly airflow (ranging from northwesterly to southwesterly)^23^ should transport the gaseous and particulate contaminants from industrial areas and urban settlements, located at the Coastal Range and Central Valley, to Andes Mountains. However, seasonal variation in winds in Central Chile may cause seasonal patterns in pollutant transport^29^. Previous studies have assessed elements in mountain snow in Central Chile resulting from urban atmospheric emissions^26^. However, to date no study has assessed the importance of atmospheric emissions in explaining the concentration of elements in high-mountain snow of central and southern Andes. In this study, we aim at assessing the relationship between air pollution and the concentrations of chemical elements along the central and southern Andean range, a *ca*. 3000‐km north–south gradient.

## Materials and methods

### Snow sampling sites

**Figure 1:**
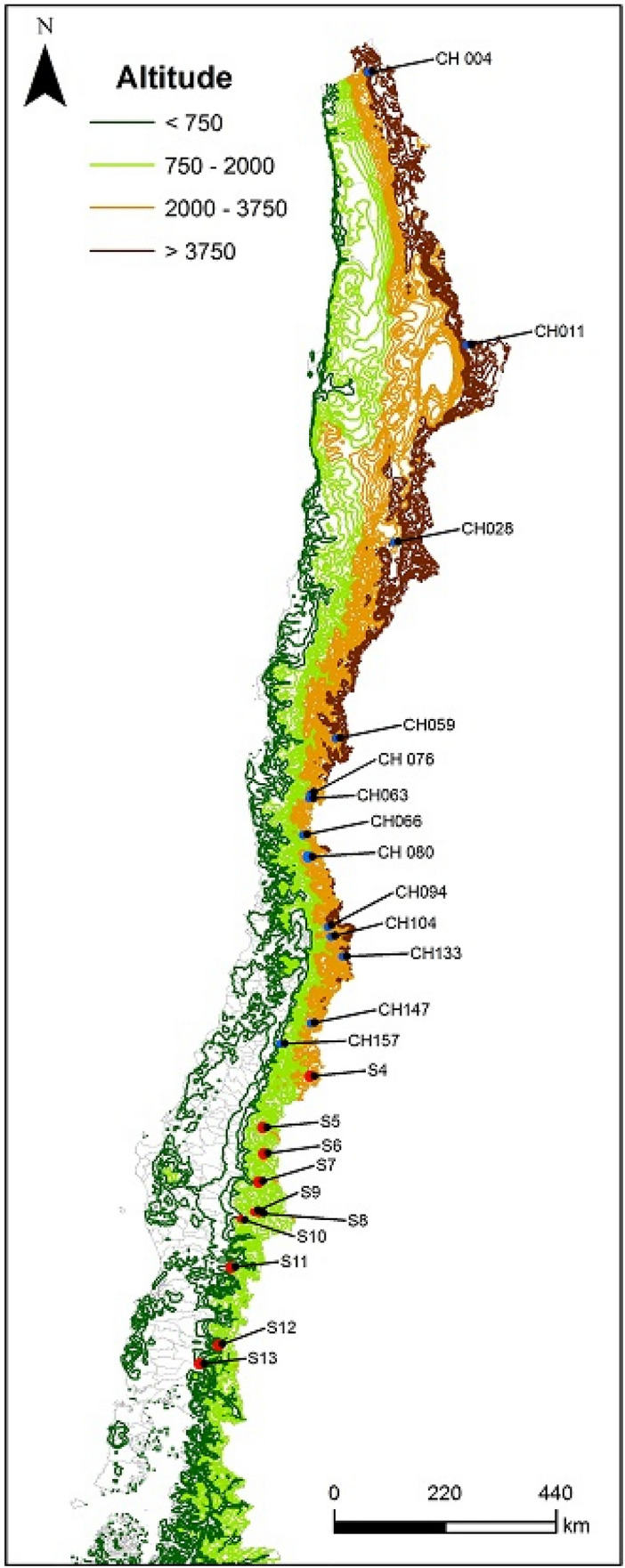
Sampling points located at high Andean mountain sites along a latitudinal transect through Chile. Attributes and code of each sampling point (northern and southern sites are blue and red points, respectively) are summarized in Table S1. Maps were developed in ArcMap v.10.2.2 (http://arcgis.com).

Field sampling was conducted in a total of 23 mountain sites along a latitudinal transect between 18°06′S (Nevados de Putre, Table S1; Fig. 1) and 41°07′S (Osorno Volcano, Table S1; Fig. 1). Snow events in the central (15–33.5°S) and southern (33.5–47°S) Andes of Chile can occur associated with frontal systems and cut-off lows that sometime can reach the northern region^23^. Convective precipitation (including snow) commonly occurs in the northernmost region of Chile during the austral summer associated with the monsoon-type circulation that develops eastward from the northern Andes^30^. Sampling sites were separated by at least 100 km and located between 1326 and 5370 m above sea level. The average altitude decreases and annual snow precipitation increases moving southward (Fig. 1). Therefore, snow depth increased moving southward as well as with increasing altitude. Sampling sites were established more than 1 km away from the nearest road, representing locations where snow accumulates during the austral winter but melts during summer. Northern sites, Nevados de Tarapacá (CH004) to Curicó (CH157), were sampled during the austral winter in July 2015 (4–27) while southern sites, Laguna del Maule (S4) to Osorno Volcano (CH157), were sampled in July–August 2016 (see site location in Fig. 1). Steep topography of sampling sites and the large distances between them hindered sampling all sites during the same year.

### Sample collection, filtering and analytical procedures

A metal spatula was used to collect snow, placed into plastic food-handling bags and stored in sealed plastic Whirlpak bags^31,32^. We obtained 500–1000 mL snow samples at depths between 5 and 80 cm, as detailed in Table S1. We were especially careful that the snow samples did not contain water derived from snow melting when collecting snow. Since in the northernmost sampling sites the depth of snow was thinner than 60 cm, samples were obtained in the first 15 cm (Table S1). From Curicó to Osorno Volcano the snow cover was deeper, and hence the snow samples were obtained from 5–15 cm and from 20–80 cm depth at locations separated by 1 m, which resulted in 33 snow sampling records (Table S1).

Bagged snow samples were kept frozen until filtration and kept at near-freezing temperatures into coolers during transportation. A glass beaker was used for melting snow, being covered to prevent external contamination. Between 200 and 1700 mL of the meltwater was vacuum-filtered (0.4 μm nucleopore filter, Whatman Nucleopore WHA10417006) with a hand or electric pump. A filter holder was mounted on a flask with as stainless-steel funnel. In order to stay within the measurement-sensitivity range of the spectrophotometer, all loadings were 0.4 to 40 µg cm^−2^. Filters were placed in Petri dishes and kept refrigerated until the analysis. The Petri dishes were opened slightly to dry to room temperature. Ultrapure water was used to clean filtration equipment after filtration. Filters containing particulate material were digested for 40 min in a microwave device (Etos One, Milestone), in teflon cups of 100 mL (Rotor SK10), with a mixture (6:2) of concentrated ultra-pure nitric acid and peroxide of hydrogen. The metal content of the resulting solution was determined in an ICP-MS equipment (Thermo Scientific X Series 2). The following elements present in the Andean snow were analyzed: Al, As, Co, Cu, Fe, Li, Mg, Mn, Na, Ni, Pb and Zn. These elements were selected based on their presence in particulate material produced from human pollution sources, including vehicular, industrial/fuel–oil combustion and secondary aerosol^33^. Particulate material from snow samples was analyzed in the Metal Laboratory of the Ecology Department of the Pontific Catholic University of Chile.

### Snow data analysis

**Figure 2:**
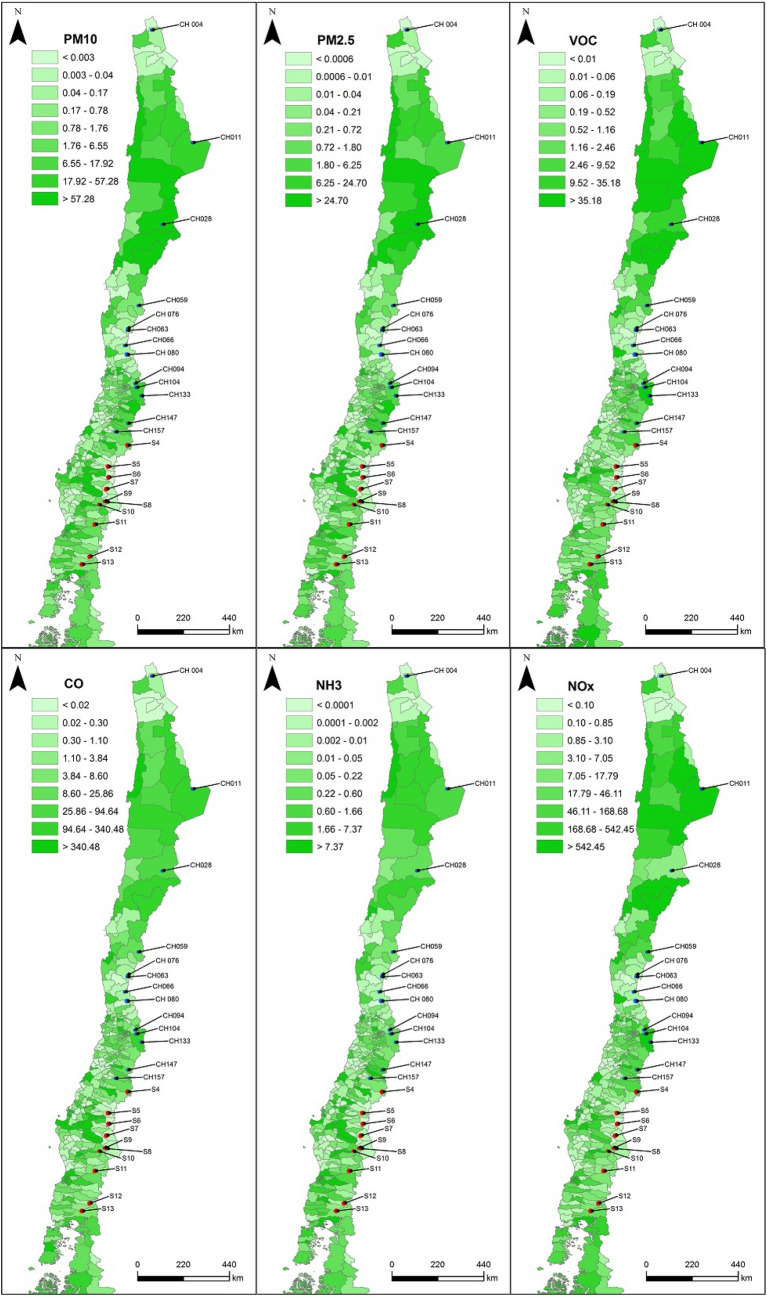
Map showing point source emissions (ton/year) of six different gaseous and particulate contaminants reported at the communal level during 2015 and 2016^35^. The code of sampling points is included as a reference. Maps were developed in ArcMap v.10.2.2 (http://arcgis.com).

Generalized Linear Mixed-effect Models (GLMM) were used to evaluate the importance of anthropogenic atmospheric emissions in the concentration of potentially toxic elements quantified in snow samples. GLMM provided a flexible framework to specify the complex error structure arising from data sampled at different regions and time periods^34^. We used inventory data of total point source emissions (ton/year) of gaseous and particulate contaminants yearly accumulated during the sampling years (2015 and 2016) provided by SNICHILE (Sistema Nacional de Inventarios de Gases de Efecto Invernadero de Chile). Emissions provided by SNICHILE database not only include those emitted in urban areas, but also pollutants derived from smelters and mining activity, particularly copper (and eventually iron). Copper mines are distributed along the entire latitudinal transect assessed in this study, concentrated in the northern and central north zones. Spatial database provides^35^ information on atmospheric contaminants measured at Chilean administrative communes, which are analogous to counties. A total of ten model predictors (atmospheric contaminants shown in Table 1 and Fig. 2) were averaged over five increasing spatial scales (10, 20, 40, 50 km around snow sampling points) at which data were available (i.e., broader spatial scales required data not available in the SNICHILE database). Specifically, around each sampling site (Table S1), contaminant emissions (*P*_*k*_) over the scale *k* were weight-averaged at buffer areas *B*_*k*_ of different radius (*k* = 10–50 km), with weights being assigned to be proportional to the area *A*_*h*_ of each commune *h* (*h* = 1 to *N*) inside the buffer area considered (Table 1), such that $P_{k}  = \sum\nolimits_{{h = 1}}^{N} {C_{h} (A_{{h,k}} /B_{k} )}$, where *C*_*h*_ is the emission reported for the commune *h*. The air mass transport of pollution within the buffer areas is mainly accounted for the prevailing westerly airflow along the study latitudinal transect^23^. Emission data of 2015 for northern points and 2016 for southern points were used as predictors in GLM.

**Table 1:** Summary of the emission rates (ton/year) of particulates and gases from anthropogenic sources estimated at different spatial scales (see main text).

Variable	Code	Mean ± SE									
		0–10 km		0–20 km		0–30 km		0–40 km		0–50 km	
Benzene	BEN	0.03	0.01	0.03	0.01	0.03	0.01	0.05	0.01	0.07	0.02
Volatile organic compound	VOC	9.36	3.05	10.25	2.86	9.15	2.58	10.56	2.58	10.47	2.47
Sulphur dioxide	SO2	7.04	2.21	7.59	2.02	7.92	1.88	22.93	6.92	51.60	23.97
Carbon dioxide	CO2	5685.3	1577.2	6403.1	1689.1	7220.0	1806.0	11,437.0	2063.0	15,214.9	2621.1
Carbon monoxide	CO	25.3	8.1	39.8	11.2	49.8	13.6	62.3	12.6	74.5	12.9
Particulate Matter (2.5 µm)	PM2.5	412.3	282.4	412.9	282.3	413.6	282.3	411.1	278.7	398.5	268.5
Particulate Matter (10 µm)	PM10	183.0	126.0	183.2	125.9	183.6	125.9	183.4	124.8	179.8	121.3
Ammonia	NH3	0.26	0.06	0.30	0.08	0.50	0.16	1.45	0.61	2.51	1.01
Nitrogen oxide	NOx	98.4	38.0	111.2	35.8	98.8	32.4	122.2	33.0	123.5	29.6
Tetra methyl benzene	TMB	0.04	0.03	0.03	0.02	0.03	0.01	0.03	0.01	0.04	0.01

Elements were standardized in order to make GLMM coefficients comparable across models, being subsequently log transformed to normalize data. Spatial autocorrelation in model residuals was controlled by including a spatial correlation structure specified by an exponentially decreasing function. In addition, a random factor was included, distinguishing between the southern and northern regions, which were sampled over different years. The Akaike Information Criterion (AIC) was used to select the most parsimonious models from the set of candidate models^36^. Most predictors were correlated with each other across different spatial scales. Thus, collinearity between predictors prevented inclusion of all predictors and spatial scales in the same models. The model selection approach involved analyzing the effect of contaminants over different spatial scales. For each spatial scale, the set of competing candidate models included all combinations of predictors that were not correlated (r < 0.5) and with a variance inflation factor (VIF) < 5. We used the dredge function, specified from the MuMIn package of the R 4.0.2 software^37^, to carry out an automated model selection using all possible combinations of predictor variables from the global model. We estimated model-averaged coefficients for the explanatory variables contained in the set of best-supported models using the model.avg function from the MuMIn R package.

## Results

The concentration of seven elements (Al, Cu, Fe, Li, Mg, Mn and Zn) was associated to gaseous and particulate contaminants emitted at different spatial scales, as shown by the best-supported candidate GLM (Table 2; Table S2). However, GLMs failed to converge for As, Co, Na, Ni, and Pb due to the concentration of those elements were very low, in some cases near to their detection limits. The concentration of Li increased significantly with altitude (Tables 2, 3).

**Table 2:** Descriptive statistics for the concentration (µg g^−1^) of chemical elements found in the snow samples (n = 39) collected at the Andes. The mean, standard error (SE) and range (Min–Max) of the concentration of each element is shown. Minimum concentrations with zero value indicate element was not detected in some points.

Element	Mean	SE	Max	Min
Al	6.54	2.13	55.44	0.01
As	0.02	0.01	0.35	0.00
Co*	0.18	0.06	2.10	0.00
Cu	0.19	0.08	2.09	0.00
Fe	6.49	2.29	42.02	0.00
K	6.48	1.76	18.39	0.20
Li	0.01	0.01	0.17	0.00
Mg	2.56	0.92	18.84	0.00
Mn	0.10	0.03	1.15	0.00
Na	0.35	0.11	2.68	0.00
Ni	0.02	0.01	0.46	0.00
Pb	0.01	0.00	0.12	0.00
Si	0.20	0.04	0.56	0.10
Zn	0.02	0.01	0.16	0.00

*Value × 100.

**Table 3:** Significant coefficients of the best-supported GLMM accounting for the concentration of trace elements in snow samples collected at the Andes. Predictors quantified at different spatial scales are: Altitude; Volatile organic compounds (VOC), Nitrogen oxide (NOx); 2.5 μm and 10 μm particulate matter (PM2.5 and PM10, respectively) and carbon monoxide (CO). Significance levels of coefficients are: *p < 0.05, **p < 0.01, ***p < 0.001.

Variable	Scale	Element						
		Al	Cu	Fe	Li	Mg	Mn	Zn
CO	20	0.057**	0.758***	0.690*		0.030***	0.172**	0.337**
CO	40	0.054**						
VOC	40							0.300**
Altitude					0.425***			
PM10	10				0.524***			
PM10	20				0.532***			
PM10	40				0.524***			
PM10	50				0.521***			
PM2.5	10				0.523***			
PM2.5	20				0.532***			
PM2.5	40				0.532***			
PM2.5	50				0.520***			
NOx	40		0.672***			0.0250***	0.132*	0.317**
NOx	50							0.300**

**Figure 3:**
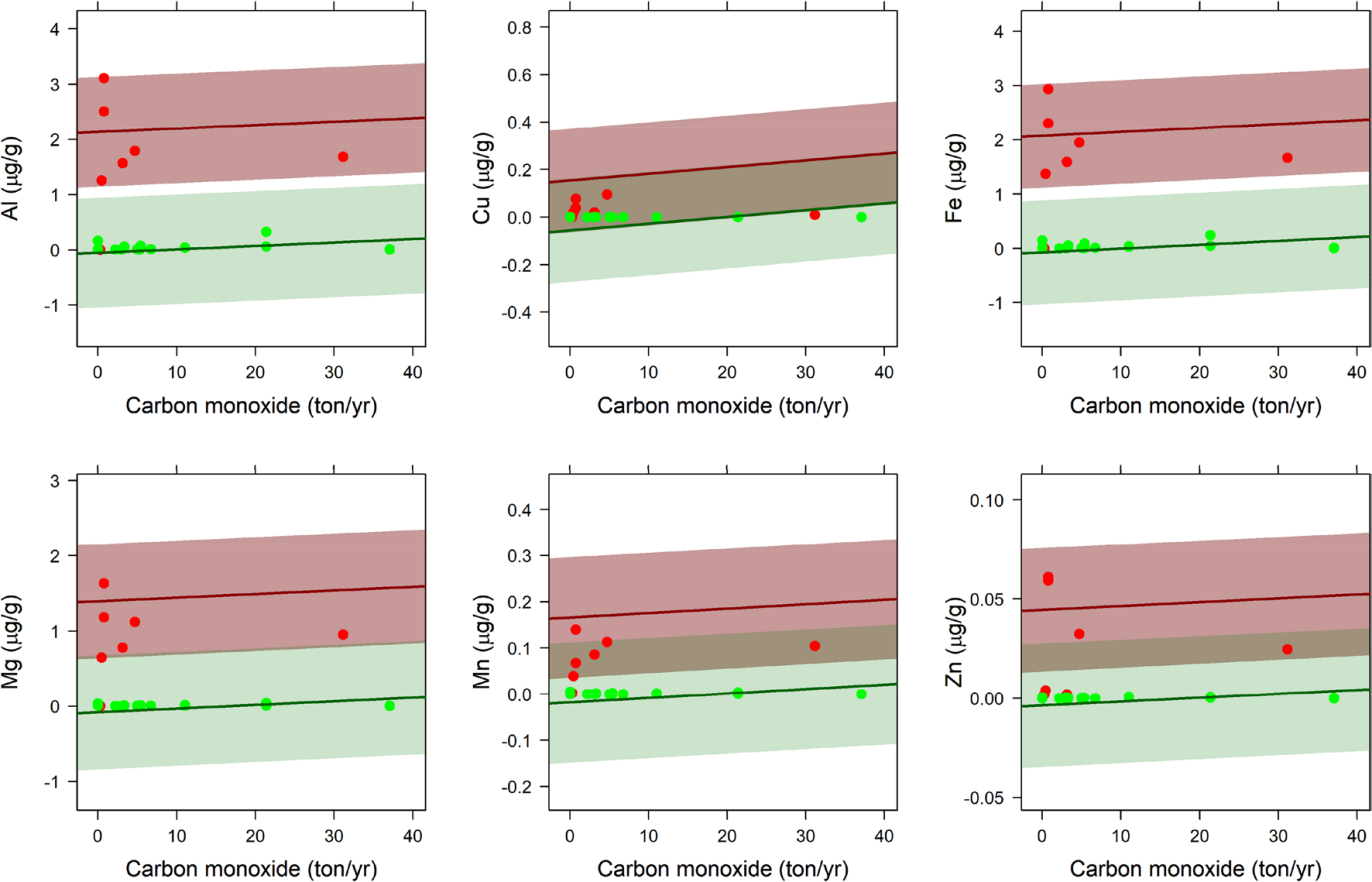
Concentration (standardized values) of trace elements as a function of carbon monoxide (CO) averaged at 20 km around sampling points, as predicted from supported GLM (Table 3). Predictions and observed values are shown separately for sampling points located at the northern (red line and points) and southern (green line and points) regions, as well as 95% confidence intervals are shadowed in red and green, respectively.

Carbon monoxide (CO) averaged at 20 km was the contaminant with the largest number of significant associations with elements (Table 3; Fig. 3). Emission of CO was positively associated to concentration of Al, Cu, Fe, Mg, Mn and Zn averaged at 20 km (Fig. 3), while CO averaged at 40 km was positively associated to concentration of Al (Table S2; Table 3). The second most important contaminant in accounting for metal concentrations was the Nitrogen oxide (NOx). Concentrations of Cu, Mn, Mg and Zn were positively associated to NOx emissions at 40 km, while NOx quantified at 50 km was associated to Zn only (Table S2; Table 3). Emission of 2.5 μm and 10 μm particulate matter (PM2.5 and PM10, respectively) averaged at 10 km, 20 km, 40 km and 50 km was positively associated to concentration of Li (Table S2; Table 3). Emission of Volatile Organic Compounds (VOC) averaged at 40 km around sampling sites was positively associated to the concentration of Zn (Table S2; Table 3). Ammonia (NH_3_) emission was not an important variable in any of the best-supported models.

## Discussion and conclusions

Air pollution from anthropogenic sources is an increasing environmental concern for mountain countries, which typically have developing economies based on the exploitation of natural resources and rapid natural to urban land conversion^38,39^. Evidences of the presence of chemical species in the snow derived from air pollution have been reported in the Himalayas^40–42^, Tibetan plateau^43,44^, Central European Mountains^45^ and Alps^46^. However, few studies have aimed at determining the impact of these contaminant emissions on natural snow reservoirs in the Andean mountains^15,47,48^. Results of our study support the idea that air contaminants emitted along a latitudinal transect are positively associated to the concentration of chemical elements measured at high-altitude mountain snow. Some contaminants were more important as contamination indicators, but also their impacts on the abundance of elements depended on the distance from the sampling site to the pollution sources, as inferred from the spatial scales at which the contaminant emissions were averaged. Previous studies suggest Zn, Pb, Cd, Ni, and Cu to be good indicators of the air pollution derived from industrial emissions, coal combustion and urban areas, with As concentration being relatively higher in urban areas^3,19^. Unfortunately, our data were not enough to analyze the concentration of As, Co, Na, Ni, and Pb. Instead, the relationships of Fe, Al, Mn, Mg, and Li with pollutant emissions can be explained as arising from the air pollution derived from the mining activities along the latitudinal gradient assessed. Increased concentration of metals near copper mining areas has been evidenced in Chile^49^, Sweden^50^, China^51^, India^52^ and Nigeria^53^, including a high concentration of Mn, Mg, Cu, Fe, and Zn. However, it is important to note that a high concentration of some of these elements (Al and Fe) could derive from their natural origin, hence they normally used to calculate enrichment factors^13,19^. When compared to natural sources, the relative contribution of anthropogenic sources to potentially toxic elements found in the snow should be higher as a site is closer to the emission points^9^. Our results suggest that latitudinal differences found in concentration of elements in the snow result from the processes involved in the incorporation of contaminants into the snow, including intensive mining activity in the northern and central north zones. In this sense, dry deposition of contaminants in the snow is highly probable in northern sampling sites (characterized by an arid climate), while wet deposition should have occurred in the rainy southern sites. In addition, sampling sites located in the northernmost areas were particularly exposed to intense solar radiation that eventually may cause snow melting faster in surface. In addition, trajectories of air masses along the study transect are dominated by the southeast Pacific Anticyclone that makes the pattern of contaminant transport to be relatively similar in all sampling sites^54^. However, local-scale patterns of wind in complex mountain terrains are known to affect the snow deposition patterns for snowfall and drifting snow^55–57^, thus, potentially modifying the spatial dispersion and distribution of pollutant particles.

Our results suggest that contaminant emissions should be considered as proxy for contaminant sources, because the spread of these contaminants probably is similar than other compounds transporting elements found in the snow. The size of the particulates^58^ that contain the potentially toxic elements may influence the distance on which those elements are dispersed. Other possible mechanism linking emissions with elements in the snow are chemical reactions among gases and particulate material (e.g., PM2.5) that result in compounds that are incorporated to the snow. Our results also indicate carbon monoxide (CO) at 20 km and nitrogen oxide (NOx) at 40 km as the main indicators of the chemical elements analyzed. In particular, the positive association of both carbon monoxide (CO) and nitrogen oxide (NOx) with Cu, Mg, Mn and Zn suggests that both contaminants share emission sources along the latitudinal transect (Fig. 2). Such associations between air contaminants and elements present in the snow were stronger at 20 km, 40 km and 50 km, suggesting that elements can travel long distances from their release points^59^. Emissions of CO, VOC and NOx in Chile are concentrated in northern Chile (20°S–30°S; Fig. 2), but also peak in Central Chile, at the mountains adjacent to Santiago, a populated city with more than 6 million people (Fig. 1). CO is originated from incomplete combustion of fossil fuel, which is usually accompanied by emission of elements present in fossil fuel^60,61^.

VOC is a precursor of particulate matter formation that may contain metals^62^, while NOx is associated with the presence of metals soluble in water^63^. In addition, snow is an efficient scavenger of contaminants^63^. Finally, we found a strong association of PM10 and PM2.5 with Li, which could emerge from a geographic convergence between intensive mining activities and presence of natural lithium sources, such as salt mines, geysers, and salt lakes located in northern Chile (22°S–26°S; Fig. 2). However, emissions of CO and NOx were not associated to Li concentration, which suggests that the presence of this element in the snow responds stronger to natural sources like dust^47^.

Our finding provides the first approach to the relation between contaminant emissions and chemical species load along the Andes Mountains. Although we have identified the best indicators for snow pollution (e.g., CO and NOx), the mechanisms responsible for these significant associations should be further identified and evaluated in order to understand physical–chemical processes of contaminant compounds in the atmosphere. The presence of elements in the snow could contribute to contamination of riparian ecosystems and glaciers. Chilean rivers at the northern regions exhibit high concentration levels of heavy metals, which have been attributed to mining activities along river basins, such as mining tailings, acid mine drainage and mining dams^64^. Indeed, some rivers of northern Chile show high concentration levels of Cu, Hg, Cd and Cr, which are particularly toxic for human consumption and agricultural use^64,65^. Since snow melting is one of the main water source for northern Chilean rivers, snow pollution (as suggested in this study) could contribute to increase the concentration heavy metals in these rivers. However, to date, no study has addressed the fraction of the toxic elements in the rivers of northern Chilean that come from the melted snow. Thus, our results contribute to expanding our understanding of the potential sources of pollution found in northern Chile Rivers and other water bodies, such as Andean wetlands^66^. Moreover, deposition of elements in Andean mountain snow of Central Chile may have an important impact on human health, as this is a densely populated region that depends on the freshwater originated from glacier and snow melting in the neighboring Andes. Recent studies have evidenced an accelerated glacier melting and decreasing snow cover in the last decades in Northern and Central Chile^67^, making human population in this region more vulnerable to limited freshwater sources. Light absorbing impurities derived from atmospheric pollution cause a darkening effect, reducing the albedo of snow and ice, as found in the remote Tibetan Plateau and Himalayas sites^4,59^. Mechanisms explaining the light-absorption by impurities in the snow of the high Andes Mountains have been found to change with latitude, being dust the main source of albedo reduction in northernmost regions^47^. In central Chile, a decrease in albedo is strongly related to particle emissions derived from vehicle traffic^68^. However, some of the elements found in the snow (e.g., Li) would form part of the chemical composition of the dust derived from lithogenic sources and deposited on the snow. Climate change also influences snow stocks in central Chilean Andes, which is evidenced by a snow albedo reduction during the 2010–2020 “Mega Drought” period^69^. Moreover, the combined effect of the distribution of black carbon and atmospheric aerosols, measured as Aerosol Optical Depth (AOD), are associated with the snow albedo decrease along the north and central Andes range^29,70^. Thus, independent from the source, an albedo reduction should increase as the load of particulate matter containing potentially toxic elements increase in the snow. In light of these developments, we expect that snow contamination from anthropogenic pollution will decrease as new environmental policies reducing mobile and point source emissions are implemented. However, our study is still preliminary in the sense of understanding the mechanisms responsible for the relationships found between chemical elements contained in the snow and atmospheric pollution emissions. We suggest future studies should consider real-time monitoring of snow chemical parameters and modeling atmospheric pollution emissions over different spatial–temporal scales.

## Supplementary Information

Supplementary Tables.
