# Supplementary material for: Contaminant emissions as indicators of chemical elements in the snow along a latitudinal gradient in southern Andes
Source: Sci Rep. 2021 Jul 15;11:14530. doi: 10.1038/s41598-021-93895-1 (PMC8282802; doi:10.1038/s41598-021-93895-1)
Supplement: Supplementary file 1 — Supplementary Tables. [file 41598_2021_93895_MOESM1_ESM.docx]

| **Site** | **Sample ID** | **Altitude (m)** | **depth (cm)** |
| --- | --- | --- | --- |
| Nevados de Tarapacá | 1 | 5318 | 0-5 |
| San Pedro: Cerro Toco | 1 | 5370 | 10-15 |
| La Ola Tranque (Copiapo) | 1 | 3624 | 10-15 |
| Valle del Elqui (El Indio) | 1 | 2341 | 0-5 |
| La Ramada (Limarí) | 1 | 1757 | 0-4 |
| Valle Choapa | 1 | 1919 | 0-3 |
| Portillo | 1 | 2800 | 10-15 |
| Los Hornos: El Juncal | 1 | 2322 | 0-5 |
| Valle Nevado | 1 | 2802 | 0-5 |
| Valle Maipo- Rio Colorado | 1 | 2302 | 0-5 |
| Valle Maipo | 1 | 2236 | 0-5 |
| Curico | 1 | 1860 | 0-10 |
| Curico | 2 | 1860 | 20-30 |
| Laguna del Maule | 1 | 1860 | 20-30 |
| Laguna del Maule | 2 | 1860 | 40-50 |
| Chillan | 1 | 1963 | 30-40 |
| Chillan | 2 | 1963 | 20-30 |
| Antuco | 1 | 1494 | 5-10 |
| Antuco | 2 | 1494 | 10-15 |
| Volcan Collaqui | 1 | 1142 | 0-5 |
| Volcan Collaqui | 2 | 1142 | 5-10 |
| Lonquimay | 1 | 1652 | 5-10 |
| Lonquimay | 2 | 1652 | 10-20 |
| Centro de ski Corralco | 1 | 1601 | 50-60 |
| Centro de ski Corralco | 2 | 1601 | 70-80 |
| Volcán Llaima | 1 | 1830 | 10-20 |
| Volcán Llaima | 2 | 1830 | 20-30 |
| Volcán Villarrica | 1 | 1450 | 0-5 |
| Volcán Villarrica | 2 | 1450 | 80-90 |
| Volcán Antillanca | 1 | 1349 | 30-40 |
| Volcán Antillanca | 2 | 1349 | 50-60 |
| Volcán Osorno | 1 | 1326 | 5-10 |
| Volcán Osorno | 2 | 1326 | 20-30 |

**Table S1.** Andean mountain sites where now sampling campaigns were carried out, including data taken during 2015 (july 4-27), from Nevados de Tarapacá (S18°06'8.5") to Valle Nevado (S 33°21.58’35.03”), and 2016 (July 13-21 and August 19 and September 1) from the Maipo Valley (33º29.8’48.6”) to Osorno Volcano (S 41°07’12.0”). Some sites had two snow samples with different depth (see sample ID).

**Table S2.** Best-supported GLM showing the additive effects of atmospheric

pollutants on chemical elements found in snow samples from Andean mountains

| **Model** | **AICc** | **ΔAICc** | **Weight** | **Element** |
| --- | --- | --- | --- | --- |
| CO (40 km) | 63.111 | 0 | 0.332 | Al |
| CO (40 km) + NOx (40 km) | 62.497 | 0.6132 | 0.2443 | Al |
| CO (20 km) | 61.586 | 1.5245 | 0.1549 | Al |
| CO (20 km) + NH_3_ (20 km) | 61.401 | 1.7093 | 0.1412 | Al |
| Masl + CO (40 km) | 61.196 | 1.9147 | 0.1275 | Al |
| Masl + CO (20 km) | 256.14 | 0 | 0.6779 | Cu |
| NOx (40 km) | 254.65 | 1.4884 | 0.3221 | Cu |
| CO (50 km) | 56.65 | 0 | 0.0865 | Fe |
| NOx (10 km) | 56.279 | 0.3716 | 0.0718 | Fe |
| CO (10 km) | 56.202 | 0.4482 | 0.0691 | Fe |
| CO (20 km) + NH_3_ (20 km) | 55.908 | 0.7423 | 0.0597 | Fe |
| NH_3_ (30 km) | 55.645 | 1.005 | 0.0523 | Fe |
| PM10 (30 km) | 55.643 | 1.0069 | 0.0523 | Fe |
| PM2.5 (30 km) | 55.643 | 1.0071 | 0.0523 | Fe |
| CO (30 km) | 55.638 | 1.0125 | 0.0521 | Fe |
| CO (20 km) | 55.605 | 1.0454 | 0.0513 | Fe |
| NH_3_ (20 km) | 55.589 | 1.0616 | 0.0509 | Fe |
| PM2.5 (20 km) | 55.489 | 1.1618 | 0.0484 | Fe |
| PM2.5 (40 km) | 55.488 | 1.1621 | 0.0484 | Fe |
| NH_3_ (40 km) | 55.488 | 1.1623 | 0.0484 | Fe |
| PM10 (50 km) | 55.487 | 1.1637 | 0.0483 | Fe |
| PM10 (40 km) | 55.485 | 1.1653 | 0.0483 | Fe |
| VOC (30 km) | 55.348 | 1.3029 | 0.0451 | Fe |
| Masl + CO (20 km) + NH_3_ (20 km) | 55.017 | 1.633 | 0.0382 | Fe |
| PM2.5 (10 km) | 55.015 | 1.635 | 0.0382 | Fe |
| PM10 (20 km) | 55.015 | 1.6355 | 0.0382 | Fe |
| Masl + VOC (50 km) + PM10 (50 km) | 93.947 | 0 | 0.0481 | Li |
| Masl + VOC (50 km) + PM2.5 (50 km) | 93.969 | 0.0222 | 0.0476 | Li |
| Masl + NOx (50 km) + PM10 (50 km) | 94.054 | 0.1074 | 0.0456 | Li |
| Masl + NOx (50 km) + PM2.5 (50 km) | 94.084 | 0.1377 | 0.0449 | Li |
| Masl + PM2.5 (20 km) | 94.148 | 0.2018 | 0.0435 | Li |
| Masl + PM10 (10 km) | 94.151 | 0.2046 | 0.0434 | Li |
| Masl + PM10 (40 km) | 94.162 | 0.215 | 0.0432 | Li |
| Masl + PM2.5 (20 km) | 94.183 | 0.2363 | 0.0427 | Li |
| Masl + PM10 (50 km) | 94.184 | 0.2376 | 0.0427 | Li |
| Masl + PM2.5 (10 km) | 94.187 | 0.2401 | 0.0427 | Li |
| Masl + PM2.5 (40 km) | 94.216 | 0.2695 | 0.042 | Li |
| Masl + VOC (40 km) + PM10 (40 km) | 94.228 | 0.2815 | 0.0418 | Li |
| Masl + VOC (40 km) + PM2.5 (40 km) | 94.247 | 0.3006 | 0.0414 | Li |
| Masl + PM2.5 (50 km) | 94.251 | 0.3046 | 0.0413 | Li |
| Masl + NOx (40 km) + PM10 (40 km) | 94.366 | 0.4193 | 0.039 | Li |
| Masl + NOx (40 km) + PM2.5 (40 km) | 94.389 | 0.4421 | 0.0386 | Li |
| Masl + VOC (20 km) + PM10 (20 km) | 94.589 | 0.6422 | 0.0349 | Li |
| Masl + VOC (20 km) + PM2.5 (20 km) | 94.598 | 0.6518 | 0.0347 | Li |
| Masl + NOx (20 km) + PM10 (20 km) | 94.709 | 0.762 | 0.0329 | Li |
| Masl + NOx (20 km) + PM2.5 (20 km) | 94.719 | 0.7728 | 0.0327 | Li |
| Masl + CO (20 km) + PM10 (20 km) | 95.413 | 1.4663 | 0.0231 | Li |
| Masl + CO (20 km) + PM2.5 (20 km) | 95.431 | 1.4843 | 0.0229 | Li |
| Masl + VOC (10 km) + PM10 (10 km) | 95.445 | 1.4983 | 0.0227 | Li |
| Masl + VOC (10 km) + PM2.5 (10 km) | 95.457 | 1.5105 | 0.0226 | Li |
| Masl + NOx (10 km) + PM10 (10 km) | 95.49 | 1.543 | 0.0222 | Li |
| Masl + NOx (10 km) + PM2.5 (10 km) | 95.504 | 1.5571 | 0.0221 | Li |
| Masl + CO (10 km) + PM10 (10 km) | 95.653 | 1.7063 | 0.0205 | Li |
| Masl + CO (10 km) + PM2.5 (10 km) | 95.667 | 1.7208 | 0.0203 | Li |
| CO (20 km) | 157.63 | 0 | 0.4722 | Mg |
| CO (20 km) + NH_3_ (20 km) | 156.99 | 0.6325 | 0.3442 | Mg |
| NOx (40 km) | 155.74 | 1.8885 | 0.1837 | Mg |
| CO (20 km) | 365.48 | 0 | 0.4383 | Mn |
| CO (20 km) + NH_3_ (20 km) | 365.28 | 0.2026 | 0.396 | Mn |
| NOx (40 km) | 363.54 | 1.9451 | 0.1657 | Mn |
| NOx (40 km) | 500.3 | 0 | 0.2126 | Zn |
| CO (20 km) | 499.69 | 0.6106 | 0.1567 | Zn |
| CO (20 km) + NH_3_ (20 km) | 499.47 | 0.8289 | 0.1405 | Zn |
| NOx (50 km) | 499.4 | 0.8926 | 0.1361 | Zn |
| VOC (40 km) | 498.76 | 1.5364 | 0.0986 | Zn |
| NOx (40 km) + NH_3_ (40 km) | 498.61 | 1.6869 | 0.0915 | Zn |
| Masl + NOx (40 km) | 498.4 | 1.8986 | 0.0823 | Zn |
| Masl + CO (20 km) | 498.38 | 1.9135 | 0.0817 | Zn |
